# Integrated metabolomics and phytochemical genomics approaches for studies on rice

**DOI:** 10.1186/s13742-016-0116-7

**Published:** 2016-03-02

**Authors:** Yozo Okazaki, Kazuki Saito

**Affiliations:** RIKEN Center for Sustainable Resource Science, 1-7-22 Suehiro-cho, Tsurumi-ku, Yokohama 230-0045 Japan; Kihara Institute for Biological Research, Yokohama City University, 641-12 Maioka-cho, Totsuka-ku, Yokohama, Kanagawa 244-0813 Japan; Graduate School of Pharmaceutical Sciences, Chiba University, 1-8-1 Inohana, Chuo-ku, Chiba 260-8675 Japan

**Keywords:** Rice, Metabolomics, Metabolism, Mass spectrometry, Phytochemical genomics, Quantitative trait loci

## Abstract

Metabolomics is widely employed to monitor the cellular metabolic state and assess the quality of plant-derived foodstuffs because it can be used to manage datasets that include a wide range of metabolites in their analytical samples. In this review, we discuss metabolomics research on rice in order to elucidate the overall regulation of the metabolism as it is related to the growth and mechanisms of adaptation to genetic modifications and environmental stresses such as fungal infections, submergence, and oxidative stress. We also focus on phytochemical genomics studies based on a combination of metabolomics and quantitative trait locus (QTL) mapping techniques. In addition to starch, rice produces many metabolites that also serve as nutrients for human consumers. The outcomes of recent phytochemical genomics studies of diverse natural rice resources suggest there is potential for using further effective breeding strategies to improve the quality of ingredients in rice grains.

## Background

Rice (*Oryza sativa* L.) is one of the most important crops worldwide and in Asian countries in particular. It serves not only as an energy source, but also as a source of nutrition. A recent report on the genomic sequencing of rice revealed that *japonica* rice was first domesticated from a population of its closely related wild ancestor (*Oryza rufipogon*) in the south part of China. Then, *indica* rice was developed by subsequent crossings of *japonica* populations with the local species of wild rice as the use of *japonica* varieties spread into southeast and south Asia [1]. As a result, there are now various rice landraces with different tastes, flavors, and tolerance to environmental stresses such as pests, drought, temperature, and nutrition limitation [2]. The metabolism of these landraces is closely related to the above mentioned traits. Thus, there have been many studies on the physiology of rice that were based on metabolomics, an approach that can be used to analyze a wide range of metabolites in each sample. Such data could greatly increase the efficacy of using the biodiversity of rice cultivars and landraces [3, 4]. Metabolomics combined with other high-throughput technology such as transcriptomics and proteomics is referred to as integrated metabolomics and is sometimes used in studies aiming to understand the metabolism as a phenotype of genome function [5, 6].

In this short review, we discuss two topics. The first is the application of metabolomics to studies aiming to provide an understanding of the association between the metabolism and certain biological events, or the metabolic changes that occur in response to interventions such as stress treatment or gene modification. The second topic is phytochemical genomics approaches to rice research. Phytochemical genomics is a recently emerging concept, the focus of which is understanding the genetic basis of phytochemical biosynthesis [7]. Rice accumulates various types of rice-specific metabolites [8], and the biosynthetic pathways that produce them are mostly still unknown. We discuss studies of rice metabolite biosynthesis based on metabolomics as a key research tool, and describe recent papers discussing metabolite quantitative trait locus (QTL) analysis.

## Review

### Use of metabolomics for the investigation of metabolism in rice

Metabolomics has often been applied to the investigation of the response to biotic or abiotic stresses in rice. For example, a metabolomic analysis of rice leaves infected with the fungus *Magnaporthe grisea,* which causes rice blast disease, presented a model for how this biotrophic/hemi-biotrophic pathogen succeeds in suppressing the host’s defenses and takes up the nutrients required to propagate in plant tissue [9] (Table 1). In this study, metabolomic analysis revealed a modification of the shikimate pathway (an increase in quinate and the accumulation of non-polymerized lignin precursors) that resulted in a reduction in lignified papillae formation and an increase of the mannitol content of susceptible hosts [9]. Since mannitol was proposed to be an important carbohydrate for fungal growth [10], its increased concentration in susceptible hosts suggests the active metabolic re-programming of infected plants by pathogens [9]. In addition, RNA-Seq and high-throughput SuperSAGE analysis based on next-generation sequencing recently revealed upregulation of quinate permease upon infection [11], which supports the data produced by the above-mentioned metabolomic study. Likewise, metabolomics integrated with transcriptomics was applied to the investigation of rice infected with *Xanthomonas oryzae*, the causal organism of bacterial leaf blight. This revealed many different metabolic responses between wild type and genetically modified rice with disease resistance [12] (The dataset for [12] is open and available at: [13, 14].

**Table 1:** Metabolomic research in rice

Category	Research materials	Analytical method	Analytes	Other omics tools	Year	Reference
Biotic stress	Leaves infected with fungal pathogen (*Magnaporthe grisea*)	Infusion-MS, GC-MS	Mainly primary metabolites, lignin monomers		2009	[9]
Biotic stress	Leaves infected with pathogenic bacteria (*Xanthomonas oryzae* pv. *oryzae*)	LC-MS, GC-MS	Mainly primary metabolites	Transcriptomics	2010	[12]
Biotic stress	Leaves of rice infected with rice brown spot fungi (*Bipolaris oryzae)*	HPLC, LC-MS	Specialized metabolites		2008	[15]
Biotic stress	Rice plants inoculated with symbiotic rhizobacterium	LC-MS	Specialized metabolites		2013	[22]
Abiotic stress	Leaves of rice challenged with submergence	^1^H NMR	Mainly primary metabolites		2012	[25]
Abiotic stress	Developing caryopses grown under high night temperature	CE-MS	Primary metabolites	Transcriptomics	2010	[28]
Abiotic stress	Leaves of rice cultivars grown under high night temperature	GC-MS	Primary metabolites		2015	[29]
Abiotic stress	Floral organs of rice cultivars under heat stress	GC-MS	Primary metabolites		2015	[33]
Abiotic stress	Leaves of rice challenged with drought stress	GC-MS	Mainly primary metabolites	Transcriptomics	2013	[30]
Abiotic stress	Leaves of rice challenged with drought stress	GC-MS	Mainly primary metabolites	Transcriptomics, proteomics	2011	[31]
Abiotic stress	Aerial parts of rice treated with cold and drought stress	GC-MS, CE-MS, LC-MS	Mainly primary metabolites	Transcriptomics	2014	[32]
Abiotic stress	Rice challenged with salt stress	GC-MS	Mainly primary metabolites		2007	[34]
Abiotic stress	Leaves of rice treated with ozone	CE-MS	Mainly primary metabolites	Transcriptomics, proteomics	2008	[35]
Abiotic stress/genetic modification	Suspension cells over-expressing cell death suppressor (BI-1)	CE-MS	Water-soluble primary metabolites		2010	[36]
Abiotic stress/ genetic modification	Leaf blade, leaf sheath, and roots of plant disrupted in glutamate synthase	GC-MS	Mainly primary metabolites		2011	[38]
Genetic modification	Grains of a double mutant rice deficient in starch synthase genes	GC-MS, LC-MS	Primary metabolites and lipids		2016	[40]
Genetic modification	High-tryptophan rice where anthranilate synthesis-related pathway is modified	LC-MS, CE-MS	Primary and specialized metabolites	Transcriptomics	2007, 2011	[41, 42]
Genetic modification	Leaves of rice expressing a moss Na^+^ transporter	GC-MS	Primary metabolites	Ionomics	2007	[43]
Genetic modification	Leaves of rice expressing NAD kinase	CE-MS	Primary metabolites		2010	[44]
Genetic modification	Leaves of rice over-expressing rice full-length cDNA	GC-MS	Mainly primary metabolites		2010	[45]
Natural variation	Grains of rice diversity research set	GC-MS, CE-MS, LC-MS	Primary and specialized metabolites, and lipids		2011	[47]
Natural variation	3 commercial rice cultivars in Laos	^1^H NMR, GC-MS, GC-MS (volatile), ICP-MS	Primary and specialized metabolites, volatiles, minerals	Genomics, ionomics	2012	[48]
Natural variation	Cooked grains of 10 rice cultivars	LC-MS	Primary and specialized metabolites	Genomics	2010	[49]
Natural variation	Grains of 51 *japonica* and 49 *indica* cultivars	LC-MS, GC-MS	Primary and specialized metabolites.		2014	[50]
Natural variation	Grains of 68 world rice core collection	GC-MS	Mainly primary metabolites		2007	[51]
Natural variation	Grains of knockout mutant disrupted in starch synthesis-related genes	GC-MS, CE-MS, LC-MS	Primarily and specialized metabolites, lipids		2012	[52]
Natural variation	Leaves of 38 rice varieties	LC-MS	Primary and specialized metabolites		2013	[62]
Natural variation	Grains of BILs	GC-MS, CE-MS, LC-MS	Primarily and specialized metabolites, lipids	Genomics	2012	[63]
Natural variation	Flag leaves and grains of 210 RILs	LC-MS	Primary and specialized metabolites	Genomics	2013	[64]
Natural variation	Leaves of 529 rice accessions	LC-MS	Primary and specialized metabolites	Genomics	2014	[66]
Natural variation	Leaves of 175 Japanese rice cultivars	LC-MS	Primary and specialized metabolites	Genomics	2015	[67]
Natural variation	Flag leaf, culm, panicle, grain, and root of 24 Chinese cultivated rice germsperm	LC-MS	Primary and specialized metabolites	Genomics	2015	[68]
Natural variation	Leaves of 322 RILs	LC-MS	Specialized metabolites	Genomics	2015	[69]

Abbreviation: *MS* mass spectrometry, *GC* gas chromatography, *LC* liquid chromatography, *HPLC* high-performance liquid chromatography, *NMR* nuclear magnetic resonance, *CE* capillary electrophoresis, *ICP* inductively coupled plasma, *BIL* backcross inbred line, *RIL* recombinant inbred line

Biotic stress or interaction with plants and other organisms greatly affect plant metabolism and sometimes the activation of specialized (secondary) metabolism can be implemented in the defense reaction against biotic stress. Metabolic profiling of rice leaves infected with rice brown spot fungi (*Bipolaris oryzae*) revealed the accumulation of serotonin with its amide conjugated with hydroxycinnamic acids [15]. The serotonin-biosynthesis-deficient mutant of rice (*sl*, Sekiguchi lesion) showed increased susceptibility to *B. oryzae* [15]*.* Serotonin is derived from the tryptophan pathway, which is often involved in the production of defensive specialized metabolites in gramineous plants (e.g., Benzoxazinone in maize, wheat, and rye [16–18]; avenanthramides in oats [19, 20]). This suggests the shared importance of the tryptophan pathway in defense-related reactions in gramineous plants [21]. Metabolic profiling has also been used to monitor strain-dependent differences in the response of specialized metabolism in rice infected with the symbiotic rhizobacterium *Azospirillum* [22].

The metabolome profile is also very sensitive to abiotic stresses. Rice often suffers from submergence, a major constraint of rice production in south and southeast Asia [23]. Adaptation to submergence in deep water is facilitated by *SUB1A*, a protein that encodes an ethylene-responsive transcription factor that restricts growth under flooding conditions [23, 24]. The metabolic profiling of the crossbred line M202 (*Sub1*) that has a higher tolerance to deep flood conditions compared to wild type M202 demonstrated that the presence of *SUB1A* in M202 led to the suppression of carbohydrate metabolism in shoot tissues [25]. This finding suggests that in the crossbred line M202 (*Sub1*) with *SUB1A*, the carbohydrate metabolism is reconstituted in a manner that suppresses elongation growth when the plant is submerged, thereby reducing energy loss under unfavorable conditions [25].

High night temperature is also a severe stress that declines yield [26] and often affects grain quality in rice [27]. Metabolomic analysis of rice grown under high night temperature conditions has been applied to find the dysregulation of central metabolism in developing caryopses (grains) [28], as well as differences in metabolic profiles among 12 cultivars with differing sensitivity to this stress during the vegetative stage [29]. In addition, metabolomic studies of rice subjected to abiotic stresses including drought [30–33], heat [33], cold [32], salts [34] and oxidative stress caused by treatment of ozone [35] and menadione (a synthetic vitamin K analog) [36] suggest that various adaptive responses could be conferred to rice via metabolic reprogramming.

Metabolomics has been also used to characterize the *in vivo* functions of metabolic rice genes. Rice possesses 3 cytosolic glutamate synthase genes that are essential to nitrogen assimilation. One of them, *OsGS1;1,* is known to be crucial to normal growth and grain filling [37], although how these isozymes diverged in the context of nitrogen assimilation process and regulation of metabolic pathways has not been well investigated. A metabolomics analysis of a mutant disrupted in *GS1;1* revealed that the disruption has pleiotropic effects on the metabolism of this mutant, which suggests that this enzyme is of physiological importance in the balancing of the metabolic network [38]. Metabolomics has also been useful in the analysis of an autophagy-deficient rice mutant *Osatg7* [39], a double mutant rice deficient in starch synthase genes *SSIIIa* and *SSIVb* (*ss3a ss4b*) [40], a high-tryptophan rice in which the anthranilate synthesis-related pathway is modified [41, 42], rice expressing a moss Na^+^ transporter [43], rice over-expressing *Arabidopsis* NAD kinase [44], and in a mutant screen for modified metabolic profiles [45, 46]. Metabolomics was also used to investigate the genetic background of quality traits in rice [47–52], the metabolic changes triggered by light and dark cycles [53, 54], and biomarkers that represent the developmental period of rice [55] (The dataset for [48] is open and available at: [56].

### Phytochemical genomics in rice

Plants synthesize many kinds of so-called specialized or secondary metabolites called phytochemicals, many of which are beneficial to humans as drugs and other health-promoting compounds. Conversely, some phytochemicals are harmful to humans and methods are required for reducing the levels of these compounds in foodstuffs. To understand the genetic basis of phytochemical biosynthesis, metabolomics is often employed in combination with QTL analysis of inbred lines and natural variants [57–60]. In this case, relatively large numbers of samples should be analyzed in order to identify the exact loci associated with such metabolic traits. Indeed, a widely targeted metabolomic approach based on a mode available in triple-quadrupole mass spectrometers called selected reaction monitoring, is likely to be a good method for assessing representative metabolites in a high-throughput manner [61, 62].

An analysis of the metabolome QTLs (mQTLs) in rice was conducted using backcross inbred lines of ‘Sasanishiki’ (high-quality *japonica* rice) and ‘Hatabaki’ (high-yield *indica* rice) to understand the genetic backgrounds associated with metabolite profile in rice grains [63]. In this study, metabolomic analysis using 4 different metabolic profiling platforms detected about 760 metabolite signals from the grains and QTL analysis identified about 800 mQTLs distributed within the rice genomes. The mQTLs acquired from datasets of 2 different harvest years clearly showed significant QTL-environment interactions in primary metabolites. In contrast, the mQTLs of specialized metabolites were detected with higher reproducibility. In the strong mQTLs, some candidate genes could also be identified via *in silico* analysis. An mQTL analysis of rice grain metabolites and flag leaves was also conducted using recombinant inbred lines derived from ‘ZS97’ and ‘MH63’, the parents of a cultivar widely grown in China [64]. This research also detected many metabolic traits and mQTLs by which the metabolic pathways, especially those for flavonoid biosynthesis, were elucidated in greater detail. Reconstitution of the corresponding metabolic pathways using genetic modification clearly demonstrated the effectiveness of mQTL analysis in the identification of unknown metabolic genes [64].

The research material used in the mQTL analysis varies from inbred lines to natural variants because the identification of single nucleotide polymorphism markers is becoming increasingly feasible thanks to the wider availability of high-throughput DNA sequencing technology [65]. Recently, a genome-wide association study (GWAS) was conducted using ~6.4 million SNPs obtained from 529 diverse rice accessions [66] and revealed substantial metabolic diversity conferred by variations in rice genomes. In this research, the contributions of 5 new genes associated with the metabolism of rice were confirmed. This also demonstrates the potential of mQTL analysis to be used as a tool in phytochemical genomics. The GWAS study also dissected the genetic architecture for generating the natural variation seen in the specialized metabolism in Japanese rice cultivars [67]. Similar approaches were also applied to determine the spatiotemporal distribution of phenolamides in rice plants and metabolome GWAS analysis identified 2 spermidine hydroxycinnamoyltransferase genes [68].

mQTL analysis has also been used to investigate the genetic background of the metabolic response of rice to stress. Metabolic profiling revealed that rice contains a non-protein amino acid, (*R*)-β-tyrosine, the concentration of which can increase in germinated seeds, leaves, roots and even exudates upon jasmonic acid treatment [69]. Genetic mapping of the β-tyrosine QTL identified the causal gene that encodes a tyrosine aminomutase. A bioassay of β-tyrosine using several dicot plants suggested that this compound plays an allelopathic role in rice [69]. These findings suggest that the investigation of biodiversity in rice cultivars and landraces could help elucidate naturally developed mechanisms for the survival of rice in various environments.

As described in rice, phytochemical genomics has been mainly used to elucidate the genes that encode biosynthetic enzymes of metabolites in leaves and grains grown under good field conditions [63, 64, 66–69]. These metabolome datasets acquired in the optimal or sub-optimal growth conditions have done well to identify many mQTLs, but many chances to understand the ecological relevance of various rice phytochemicals might have been lost because some metabolic pathways can only be activated in response to biotic and abiotic stress. More in-depth mQTL analysis of rice grown under various stress conditions would reveal the hidden functions of rice genomes in the adaptation to various growth conditions, although this would not be a trivial task. A combination of mQTLs and information in databases of QTLs regarding various agronomic traits [70] could serves as a reference for further studies on the ecological relevance of various rice phytochemicals.

Identification of the function of genes related to the metabolite biosynthesis is still difficult and time-consuming. Introduction of genes of interest into rice itself or other model plants [71] and reverse genetics [72–74] have been used to confirm the gene functions *in vivo*. A technology for targeted gene mutagenesis in plants including rice is rapidly developing [75, 76], suggesting that the precise elimination of gene function in rice will be more facile in the future. In addition, rapid and space-saving rice breeding systems that enable researchers to drastically shorten the life cycle of some cultivars have been developed [77]. A combination of these technologies will help to accelerate the phytochemical genomics in rice.

Metabolomics has provided irreplaceable information on rice metabolism. The techniques for data recording and processing of metabolomics are more sophisticated than ever. Thus, it may be possible to focus efforts on validating various hypotheses elucidated from existing metabolomics research. Metabolomics has long functioned as a “hypothesis generator” [78] and these hypotheses remain to be assessed in further studies.
